# The think aloud paradigm reveals differences in the content, dynamics and conceptual scope of resting state thought in trait brooding

**DOI:** 10.1038/s41598-021-98138-x

**Published:** 2021-09-30

**Authors:** Quentin Raffaelli, Caitlin Mills, Nadia-Anais de Stefano, Matthias R. Mehl, Kate Chambers, Surya A. Fitzgerald, Ramsey Wilcox, Kalina Christoff, Eric S. Andrews, Matthew D. Grilli, Mary-Frances O’Connor, Jessica R. Andrews-Hanna

**Affiliations:** 1 Department of Psychology, University of Arizona, 1503 E University Blvd., Tucson, AZ 85721 USA; 2 Department of Psychology, University of New Hampshire, Durham, NH USA; 3 Department of Psychology, University of British Columbia, Vancouver, BC Canada; 4 Centre for Brain Health, University of British Columbia, Vancouver, BC Canada; 5 Department of Neurology, University of Arizona, Tucson, AZ USA; 6 Evelyn F. McKnight Brain Institute, University of Arizona, Tucson, AZ USA; 7 Cognitive Science, University of Arizona, Tucson, AZ USA

**Keywords:** Human behaviour, Psychology

## Abstract

Although central to well-being, functional and dysfunctional thoughts arise and unfold over time in ways that remain poorly understood. To shed light on these mechanisms, we adapted a “think aloud” paradigm to quantify the content and dynamics of individuals’ thoughts at rest. Across two studies, external raters hand coded the content of each thought and computed dynamic metrics spanning duration, transition probabilities between affective states, and conceptual similarity over time. Study 1 highlighted the paradigm’s high ecological validity and revealed a narrowing of conceptual scope following more negative content. Study 2 replicated Study 1’s findings and examined individual difference predictors of trait brooding, a maladaptive form of rumination. Across individuals, increased trait brooding was linked to thoughts rated as more negative, past-oriented and self-focused. Longer negative and shorter positive thoughts were also apparent as brooding increased, as well as a tendency to shift away from positive conceptual states, and a stronger narrowing of conceptual scope following negative thoughts. Importantly, content and dynamics explained independent variance, accounting for a third of the variance in brooding. These results uncover a real-time cognitive signature of rumination and highlight the predictive and ecological validity of the think aloud paradigm applied to resting state cognition.

## Introduction

Contemplated upon by early Buddhists and later by William James, the stream of thought, or consciousness, is an obscure but ubiquitous aspect of our waking life^1^. Although dynamics are a central feature of conscious experience, static methodologies such as retrospective recall and experience sampling have dominated our mainstream understanding of human thought. As a result, relatively little is known about the way thoughts arise and unfold over time, especially during idle time when left to our own musings^2^. The dynamics of thought may offer unique insight into the relationship between mental life and mental health. Indeed, although repetitive thinking may be beneficial at times^3,4^, rumination and worry are both characterized by *maladaptive repetitive thinking*, a transdiagnostic construct fueling maladaptive outcomes across multiple diagnostic categories^5,6^. Rumination in particular (especially the brooding facet) may signify a maladaptive form of coping involving negative mood with detrimental effects on the onset, length, and severity of depressive symptoms^7–9^.

According to our Dynamic Neurocognitive Framework, rumination can be considered a form of non-deliberate thinking guided by high “automatic” constraints that limit the conceptual scope and dynamics of thought^10–12^. Automatically constrained thoughts may feel “sticky” because they are pulled by salient affective or sensory information and, as a result, may be difficult to disengage from. An important feature of automatic constraints is their potential to differentiate rumination from spontaneous forms of thinking^13^-such as mind-wandering and dreaming—that move freely over time without constraints to guide them^11,12,14,15^. Whereas ruminative thinking may worsen mood at subsequent timepoints^16,17^, freely-moving thoughts are associated with positive subsequent mood^18^.

Despite theories highlighting the repetitive and inflexible nature of automatically-constrained cognition, relatively little empirical work has quantified the dynamic trajectories of naturally-occurring thoughts in ruminative and non-ruminative individuals. Sticky thoughts could manifest as thoughts of longer duration, as shorter thoughts that resurface more frequently over time, or both. How the affective content of thought impinges upon its duration and recurrence also remains to be delineated in both ruminative and non-ruminative individuals.

Of relevance is the concept of *emotional inertia*, the propensity to maintain one’s affective state from one moment to the next^19^. Emotional inertia is conceptualized as a maladaptive lack of emotional reactivity to the environment, where one’s current emotional state is maintained despite situational changes in which a different emotional response would be more adaptive^19^. Interestingly, higher trait rumination tends to predict stronger emotional inertia for both negative and positive emotions^20^, though negative emotional inertia is stronger in depression^20,21^. Although emotional inertia characterizes affective as opposed to cognitive inflexibility, emotional inertia theories may be relevant for conscious thought because individuals who tend to get “stuck” affectively may be more likely to get stuck cognitively.

Other theories, including the Broaden-and-Build theory of Fredrickson and colleagues^22,23^, suggest that positive and negative affect may have opposite effects on cognitive flexibility, with the former sparking more flexible and exploratory forms of cognition, and the latter leading to more rigid forms of cognition. This tendency may be self-perpetuating, creating upward or downward spirals of cognitive-affective states^23^ which may be exacerbated in ruminative individuals. In their Attentional Scope Model of Rumination, Whitmer and Gotlib^24^ propose that the array of thoughts, percepts, and actions may be inherently narrower in individuals who ruminate more frequently, regardless of whether ruminative individuals are experiencing positive or negative moods. This narrowing of attention aligns with findings that individuals with high trait rumination can exhibit stronger maintenance of task-relevant goals, leading to better performance on executive function tasks emphasizing cognitive stability^25^. Understanding how trait rumination alters the dynamics and conceptual scope of thought is a major goal of the current study.

Notably, the clinical relevance of cognitive rigidity may be amplified when individuals are left to the mercy of their thoughts for extended periods of time, especially without an external task or activity to distract themselves. Indeed, in early sensory deprivation studies, some participants reported difficulty being alone with their thoughts and began to “mind wander in a disturbing fashion”^26,27^, findings convergent with a recent empirical study^28^. In more typical contexts, excessive down time for ruminative individuals may allow automatic modes of thinking to resurface, which may exacerbate negative affect and trigger a further narrowing of the scope of attention. Interestingly, this may be one reason behind why ruminative individuals have faced particular mental health challenges during the COVID-19 pandemic^29,30^. Understanding the conscious experience of individuals during times in which they have nothing but their thoughts for distraction may thus have important clinical relevance in differentiating between those for whom internal life is enriching from those for whom it is destructive.

### The present study

Quantifying the stream of consciousness for naturally occurring, unprompted thoughts requires a paradigm that captures as much as possible the entirety of thought content in contexts where thoughts are not constrained by experimental task demands. First used to study the thought process of problem solvers and experts^31,32^, the think aloud paradigm (TAP) has been used sporadically over the past 50 years to investigate comprehension tasks^33,34^, autobiographical memory and future thought^35–38^, confabulation^39^, and more recently, unprompted thoughts^40–42^. Here we extend beyond prior studies by adapting the oral TAP to explore the basic dynamic properties and ruminative correlates of unprompted thoughts at rest in healthy adults.

Across two studies, we audio recorded participants as they spoke aloud the contents of their conscious experience for 10 min while alone in an unstimulating experimental testing room. Study 1 aimed to assess the validity of the think aloud resting state paradigm and to quantify the basic content and dynamic characteristics of thought in young adults as quantitative benchmarks for Study 2. Motivated by the Broaden and Build theory^22,23^, we predicted that thoughts rated as more negative would lead to a narrowing of conceptual scope over time. Study 2 aimed to test the replicability of results from Study 1 and to explore relationships with individual differences in brooding, a maladaptive subtype of rumination^7,9^. We hypothesized that higher brooding scores would be associated with more internal, negative, past-oriented, and self-focused thoughts, as well as restricted dynamics and a narrower conceptual scope. Based on the inconsistencies in the literature reviewed above, we were conflicted as to whether thoughts would demonstrate restricted dynamics for negatively-valenced thoughts alone, or whether restricted dynamics with greater trait rumination might also extend to positive thoughts.

## Study 1

### Study 1 methods

#### Participants

Thirty-three University of Arizona undergraduates participated in Study 1 for monetary compensation (n = 12) or research credit (n = 21). Written informed consent was obtained from all participants and all procedures were performed in accordance with the relevant guidelines and regulations and approved by the University of Arizona’s Institutional Review Board. Five participants had difficulty understanding the task and did not complete the task as instructed. A computer malfunction prevented data collection for one additional participant. The total analyzed sample thus included 27 participants (13 female, mean age = 19.12 years, range = 18–22 years; see Supplementary Table S1 for demographics).

### Think aloud paradigm

**Figure 1:**
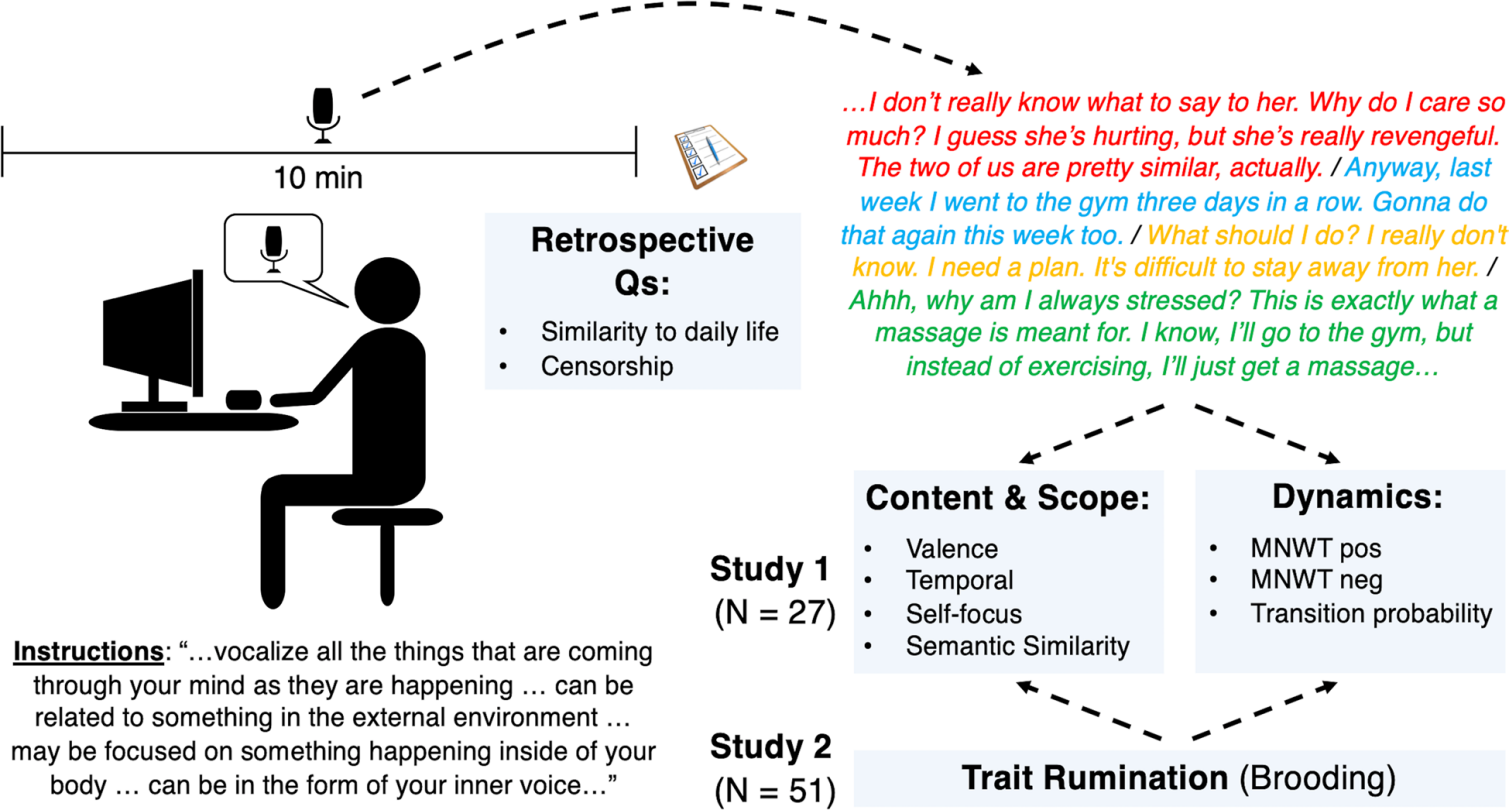
Think aloud paradigm. Participants were audio recorded while voicing aloud their unprompted thoughts for 10 min. Audio recordings were transcribed and coded by hand or automated text analysis for content and dynamics. These indices were explored as predictors of individual differences in trait brooding. MNWT: Mean number of words per thought.

We used an adapted version of the TAP to audio record participants’ continuous stream of consciousness as individuals sat alone in a testing room for 10 min (Fig. 1). Similar to Samson et al.^40^, Sripada and Taxali^41^, and Van Calster et al.^42^, participants were not prompted what to think about. Unlike these studies, participants sat alone in a normally lighted testing room. Similar to these studies, there was minimal external stimulation and the sole instruction was to continuously voice aloud whatever came to their mind, including internal thoughts or images, any perceptions of external stimuli, or any bodily sensations or feelings such as aches, pain, or hunger (similar to a written version of the task in Pennebaker and King^43^). See Supplementals for complete instructions.

Following the task, participants answered using a continuous sliding scale “How similar were your thoughts to those you experience in your day-to-day life?” and “To what extent did you censor yourself during the task?”. The cursor’s default position was the midpoint between “0-Not at all” and “1-Extremely”. The final location of the cursor was turned into a two-decimal number between 0.00 and 1.00. Participants answered additional questions and completed additional tasks which will be the focus of future manuscripts.

### Quantifying individual thoughts, transitions, and themes

Audio files were first transcribed, and three trained independent raters then partitioned the transcript into individual thoughts separated by *strong* or *associative transitions*. Thoughts separated by a strong transition demonstrated no obvious content relationship with each other, and the transition felt abrupt (e.g., “*Um, hopefully I’ll get the new job ‘cause I really want it. I’m tired of my old job.* [strong transition] *Um, I miss my dog. I haven’t seen her in a long time*.”). Associational transitions occurred when two thoughts were related in some aspect of content, but nonetheless pertained to different overarching themes, epitomized by the expression “this reminds me of …” (e.g., *“Yeah I had Japanese subtitles. I preferred Japanese rather than English. Just sounds very whitewashed when it’s in English* [associational transition] *But speaking of Japanese, I’m glad I was able to choose what I wanted to do regarding my other language.”)*.

This coding scheme provided us with: (a) *total word count* (overall verbal fluency or the rate with which thoughts are formed and vocalized), (b) *total number of thoughts* generated (fluency at the level of individual thoughts), (c) *total number of strong transitions*, (d) *total number of associational transitions*, and (e) *mean number of words averaged across all thoughts* (MNW_all thoughts_, a dynamic estimate of thought duration). Mean number of words per thought was also estimated separately for positive, neutral, and negative thoughts (see below). Raters’ scores were averaged to obtain a mean rating for each variable. Inter-rater reliabilities for each measure ranged from moderate to excellent, with the exception of associational transitions for which it was poor (see Supplementary Table S2).

### Assessing thought content via manual ratings

After individual thoughts were delineated, each thought was manually rated by two trained independent raters on several content variables. *Perceptual orientation* was assessed by characterizing each thought as predominantly externally-focused, internally-focused, or interoceptive in nature. Each thought was also manually assigned a predominant *temporal orientation* (past, present, future, or atemporal), and an overall *valence* from -5 (extremely negative) to 5 (extremely positive), with 0 = neutral. Degree of *self-focus* was rated manually for each thought using a scale from 0 (thinking about something entirely unrelated to one’s self) to 4 (clearly and objectively focusing on oneself).

### Assessing conceptual scope with semantic similarity

One aspect of dynamics may be captured by how thoughts shift in conceptual scope over time. We thus used natural language processing techniques to estimate the scope of thought using semantic similarity metrics. More similar thoughts likely reflect a narrower conceptual scope over time while less similar thoughts likely reflect a subsequent broadening of thoughts. Using the transcripts manually coded for valence, we computed two metrics of semantic similarity using the spaCy library in Python (https://spacy.io/), one of the fastest and most accurate natural language processing tools available^44,45^. *Adjacent similarity* captures the “movement” between thoughts, or how similar a given thought (thought *t*) is to the next thought (thought *t* + *1*). Given that people may return to general topics outside of immediate transitions, we also computed *average subsequent similarity*, the average similarity between thought *t* and each subsequent thought (i.e., average [similarity t_1_ − t_2_, similarity t_1_ − t_3_, … similarity t_1_ − t_final thought_]). We computed semantic similarity metrics (ranging from 0–1) using a large vector model from spaCy (685 k unique vectors) that derives a “distance” between two thoughts using their mathematical likeness (e.g. “truck” is semantically similar to “car”), after removing unmeaningful “stop” words. Such likeness is determined based on previously established relationships built on text from the web (blogs, news, comments, etc.) and is recommended when a large vocabulary is relevant, as is likely the case for thought data.

## Study 1 results

### The think aloud paradigm provides an ecologically-valid measure of everyday thinking styles

Participants reported not to censor themselves very much (*M* = 0.28, *SD* = 0.20) and experienced thoughts that were fairly similar to those in their daily lives (*M* = 0.69, *SD* = 0.23). On average across participants, the 10-min task yielded 1,216 words (*SD* = 395), in line with what we should expect if participants completed the task as instructed. Considering that speech production for an English speaker is approximately 6 syllables per second^46^ and a typical spoken word has a mean of 1.35 syllables^47,48^, we can expect 10 min of continuous speech without pauses to yield 2,650 words. However, most humans insert pauses between distinct thoughts and sentences. Participants generated a mean of 28 thoughts (*SD* = 15), comparable to that reported in Sripada and Taxali^41^, with a mean length of 60 words,. Strong transitions represented about two-thirds (*M* = 0.66, *SD* = 0.21) of all transitions for each participant. Results are reported in Table 1. In sum, these findings suggest that the TAP has strong ecological validity.

**Table 1:** Ecological validity and thought characteristics in Study 1 and 2.

	Study 1			Study 2			Study 1–Study 2 differences	
	Mean	SD	Median	Mean	SD	Median	Significance test	*P*-value
**Experience of the task**								
Thought censorship	0.27	0.20	0.25	0.40	0.27	0.40	t = − 2.43	0.012*
Similarity to everyday life	0.69	0.23	0.68	0.74	0.19	0.75	W = 608.50	0.40
**Thought characteristics**								
Total word count	1215.67	395.32	1161	1284.73	356.40	1246	t = − 0.76	0.45
Total # of thoughts	28.48	15.18	24	29.35	13.68	30	t = − 0.25	0.80
Total # of strong transitions	20.19	15.84	17	20.02	12.66	18	W = 659	0.76
Total # of associative transitions	7.22	3.77	8	8.31	5.36	7	t = − 1.05	0.30
MNW_all thoughts_	60.13	47.96	47.12	60.31	51.16	44.41	W = 705	0.87
MNW_positive thoughts_	69.46	59.61	51.39	60.19	42.28	42.10	W = 593	0.70
MNW_negative thoughts_	68.30	51	43.40	69.20	60.74	46.75	W = 691	0.98

Statistical differences between Study 1 and 2 were evaluated with a two-tailed Welch t-test for normally distributed variables and a Wilcoxon rank-sum test for non-normally distributed variables. The scale of the thought censorship and similarity to everyday life questions is ‘0-Not at all’ to ‘1-Extremely’. *MNW* Mean Number of Words. Highlighted in bold are statistically significant group comparisons at p < .05. *P < 0.05.

### Content benchmarks are similar to prior studies of everyday thought

As shown in Table 2, manual ratings of perceptual orientation revealed that 73% (*SD* = 17%) of participants’ thoughts were internal/imaginative, 24% (*SD* = 15%) were external/perceptually-coupled and 3% (*SD* = 3%) pertained to interoceptive experiences such as drowsiness, itch, and hunger, similar to Van Calster et al.^42^. Thoughts were most often rated as present-oriented (*M* = 50%, *SD* = 21%), followed by future-oriented (*M* = 21%, *SD* = 18%), past-oriented (*M* = 17%, *SD* = 11%), and atemporal (*M* = 12%, *SD* = 10%). However, the percentage of future-oriented thoughts increased in frequency when internal thoughts were analyzed alone (present: *M* = 31%, *SD* = 27%; future: *M* = 30%, *SD* = 26%; past: *M* = 23%, *SD* = 22%; atemporal: *M* = 16%, *SD* = 16%). On average, and in line with prior studies^49^, thoughts were rated as predominantly neutral in valence (on scale of − 5 to 5, *M* = − 0.15, *SD* = *0.4*7). A one sample t-test determined this rating was not statistically different from 0 (*t*(26) = − 1.67, *P* = 0.11, *CI*_*95*_: [− 0.34; 0.04], *Cohen’s d* = 0.32). Thoughts were also rated as moderately self-focused (*M* = 2.18, *SD* = 0.72, on a scale of 0 to 4).

**Table 2:** Content characteristics in Study 1 and 2 assessed manually by rater (top) and with Linguistic Inquiry Word Count (LIWC) software (bottom).

	Study 1			Study 2			Study 1–Study 2 differences	
	Mean	SD	Median	Mean	SD	Median	Significance test	*P*-value
**Thought Content: Manual ratings**								
% Internal	73%	17%	74%	74%	20%	76%	W = 674	0.88
% External / Perceptually-coupled	24%	15%	23%	23%	19%	21%	W = 727.50	0.69
% Interoceptive	3%	3%	1%	3%	4%	1%	W = 683.50	0.96
% Past	17%	11%	17%	17%	15%	12%	W = 782.50	0.33
% Present	50%	21%	50%	45%	22%	44%	W = 755.50	0.48
% Future	21%	18%	17%	27%	15%	25%	W = 519	0.076
% Atemporal	12%	10%	10%	11%	12%	8%	W = 776	0.36
Valence (-5 to 5 scale)	− 0.15	0.47	− .0.16	− 0.18	0.81	− 0.09	t = 0.20	0.84
Self-focus (0 to 4 scale)	2.18	0.72	2.13	2.18	0.61	2.21	t = − .0.05	0.96
**Thought Content: Linguistic analysis with LIWC**								
% Use of personal pronoun “I”	9.72	2.74	9.55	9.75	2.78	9.93	t = − .0.05	0.96
% Positive emotion words	2.63	1.19	2.46	3.09	1.44	2.81	t = − 1.50	0.14
% Negative emotion words	1.78	1.06	1.71	1.75	.96	1.52	t = .0.10	0.92
% Past-related words	3.41	1.31	3.49	3.04	1.2	2.89	t = 1.23	0.23

Statistical differences between Study 1 and 2 were evaluated with a two-tailed Welch t-test for normally distributed variables and a Wilcoxon rank-sum test for non-normally distributed variables. *MNW* Mean Number of Words.

In summary, thoughts emerging during the TAP tended to be predominantly internal in orientation, moderately self-focused, neutral in valence, and mostly present and prospective in nature. These findings are generally in agreement with prior literature using retrospective questionnaires to assess phenomenological characteristics of thoughts during resting state paradigms^49–52^.

### Increased negative valence leads to a narrowing of conceptual scope

Separate linear mixed effect models were constructed to assess the relationship between adjacent or average subsequent semantic similarity and manual ratings of valence for each thought. For these models, manually-rated valence was the dependent variable, and semantic similarity was the fixed effect of interest. We also included a random intercept to allow for baseline variability in valence across participants. When including random slopes in our model, they did not converge, suggesting our data does not support this more complex model structure.

The relationship between adjacent similarity and valence was statistically significant (*b* = − 0.59, *χ*^*2*^(1) = 4.78, *P* = 0.029), revealing a generally negative pattern (*ß* = − 0.08 (CI_95_: [− 0.17; − 0.01]). As thoughts became more negative, they became more semantically similar to the next thought. Conversely, as thoughts became more positive, they became more semantically distant to the next thought. Similar findings were observed with the average subsequent similarity metric (*b* = − 1.12, *χ*^*2*^(1) = 9.49, *P* = 0.002, *ß* = − 0.16 (CI_95_: [− 0.26; − 0.06]), where more negative thoughts were more semantically similar to the average of all subsequent thoughts. These findings suggest negative thoughts are associated with a subsequent narrowing of conceptual scope while positive thoughts are associated with a subsequent broadening of conceptual scope.

## Study 1 discussion

Overall, the results of Study 1 make three important contributions to the literature. First, most participants did not appear to experience the TAP as overtly artificial, and were able to experience and vocalize thoughts that were fairly representative of those experienced in daily life. Thought content was also in general agreement with the prior literature; thoughts were moderately self-focused, neutral in valence, and mostly pertained to the present and future. Beyond content, the TAP offers additional indices of dynamics, including the overall number and length of thoughts that can be explored separately for positive and negative thoughts. Third, although most thoughts were characterized by abrupt transitions, the conceptual similarity or scope of thoughts over time is moderated by thought valence. More negative thoughts were associated with a subsequent narrowing of the conceptual scope of attention.

To test the replicability of these results and examine how stream of consciousness indices vary by trait rumination, we conducted a second study using identical procedures as Study 1. Three important additions to Study 2 were: 1) the measurement and analysis of individual differences in trait brooding, 2) content coding of thoughts using automated text-analysis software, and 3) the computation of affective transition probabilities between affective thoughts.

## Study 2

### Study 2 methods

#### Participants

Fifty-seven University of Arizona undergraduates with high English proficiency participated in Study 2 in exchange for class credit. Written informed consent was obtained from all participants and all procedures were performed in accordance with the relevant guidelines and regulations and approved by the University of Arizona’s Institutional Review Board. Six participants were excluded as a result of failure to comply with the instructions of the task (n = 3), poor sound quality (n = 1), and a computer malfunction in which data were not collected (n = 2). The final sample consisted of 51 participants (32 females, 1 non-binary, *M* = 19.78 years, range = 18–28 years). Demographic details are listed in Supplementary Table S1.

#### Materials and procedures

Participants in Study 2 completed an identical TAP as Study 1 in the same room and with identical instructions. The audio files were transcribed in the same way and by the same researchers as in Study 1. To estimate individual differences in trait rumination, participants also completed the Rumination Response Scale (RRS)^9^. As we were most interested in the dynamics of maladaptive rumination^9^, we used scores from the 5-item RRS-brooding subscale for subsequent analyses^53^ which has good reliability (α = 0.77)^9^. Participants’ brooding scores ranged from 6 to 16, covering much of the scale (5 to 20) (*M* = 10, *SD* = 2.93).

A key aim of Study 2 was to determine the replicability of results from Study 1; therefore, we implemented identical data preparation and analysis procedures. The intra-class correlation coefficients for variables measured across the two studies were similar (see Supplementary Table S2) and remained within the same interpretative category (e.g. excellent, good, moderate, poor).

#### Automatic text analysis

In addition to manual coding, content was also assessed automatically using one of the most widely used and extensively validated dictionary-based text-analysis applications: Linguistic Inquiry Word Count (LIWC) v.2015^54,55^. LIWC coding offered ease and rapidity of implementation and allowed us to estimate its potential for scalability in future studies. LIWC calculates the proportion of words in the transcripts belonging to a given number of linguistic categories. Here we focused on the proportion of positive, negative, past-oriented, and future-oriented words used, as well as self-focus assessed with first person pronoun usage (e.g. I, me, my), which has reliably been linked to depression and negative emotionality^56^.

#### Statistical analysis

Partial correlations between brooding scores and indices spanning content and dynamics were run, controlling for individual differences in perceived censorship and similarity of thoughts to daily life (for first order Pearson correlations, see Supplementary Table S3). For models including dynamic indices, analyses additionally controlled for total word count. One data point with high leverage (Cook d > 0.50) was removed for two analyses (manually-coded past and MNW_all thoughts_). No analysis violated assumptions for linear modelling analysis.

As in Study 1, content analyses focused on perceptual orientation, valence, self-focus, and temporal orientation. For dynamics, we examined relationships between trait brooding and the total number of thoughts, as well as three duration indices: MNW_all thoughts_, MNW_negative thoughts_ and MNW_positive thoughts_ (though we also report MNW_neutral thoughts_ for completeness). Each thought was classified as positive when its average valence rating across the two raters was ≥ 1 (on a scale of − 5 to 5), negative when its valence rating was ≤ − 1, and neutral when its rating was [− 0.99 to 0.99]. We tested whether the various benchmarks measured in Study 1 differed in Study 2 with two-tailed Welch t-test or Wilcoxon rank-sum test depending on whether the variable distribution were normally distributed as determined by a Shapiro–Wilk test. The Welch t-test was used for all Study 1 vs. Study 2 comparisons that did not violate the distribution normality assumptions because it controls for Type 1 error better than the Student’s t-test in case of unequal variances between the groups and has been shown to be just as robust when this assumption is met^57^.

We also repeated the analyses from Study 1 to determine whether negative valence was once again related to the conceptual scope of subsequent thoughts. In light of our hypotheses, we also assessed whether brooding scores moderated this relationship.

In addition, we calculated affective transition probabilities; the likelihood that for each thought of a particular valence (Positive, Neutral, Negative), the next subsequent thought will remain in the same valence state (e.g. Pos → Pos, Neut → Neut, Neg → Neg) or shift to one of the other states (e.g. Pos → Neut, Pos → Neg, etc.). Affective transition probabilities were calculated separately across all positive thoughts, all neutral thoughts, and all negative thoughts. This method resulted in a 3 × 3 table with all possible transitional states (see Supplementary Table S4 for descriptive statistics).

Finally, we performed nested model comparisons to determine whether the dynamic indices explained variance in brooding scores above and beyond that explained by content alone. We compared a) a baseline model with all significant content variables (separate models for manual and automated coding), censorship, and daily thought similarity, to b) an intermediate model adding the total word count (to account for the contribution of verbal fluency), to c) a final model including content, censorship, daily thought similarity, total word count, and all significant dynamic variables significantly related to brooding scores (MNW_positive_, MNW_negative_, and affective transition probabilities from Pos → Pos and Neg → Pos).

### Study 2 results

#### Study 2 replicated the findings of Study 1

All but one of the indices explored in Study 1 were statistically similar to those obtained in Study 2 (Tables 1 and 2). Participants reported censoring themselves during the task more in Study 2 than Study 1 (*t*(67.62) = − 2.43, *P* = 0.012, *Cohen’s d* = 0.53). Also, there was a noticeable though non-significant increase in future orientation in Study 2 (*P* = 0.076). Restricting the temporal analysis to internally generated thoughts, future orientation became the most prominent temporal orientation in Study 2 (39% future, 23% present, 17% past, 12% atemporal). In summary, results from Study 2 largely mimic those from Study 1, suggesting the task is ecologically-valid and reliable across two independent samples.

#### Trait brooding was associated with more negative, past oriented, and self-focused thinking

As hypothesized, high brooding scores were associated with thoughts that were more negative and past-oriented for both manual ratings and automated text analysis (all *p*’s < 0.04, Table 3). In line with our predictions, trait brooding was also linked to a higher frequency of first-person pronoun usage. However, manual ratings of self-focus did not relate to trait brooding (*partial correlation* = 0.19, *P* = 0.19).

**Table 3:** Thought content and dynamic correlates of trait brooding.

Content					
LIWC	Partial r (p)	CI_95_	Manual coding	Partial r (p)	CI_95_
% Positive words	− .0.4 (0.34)	[− .0.40; − 0.14]			
% Negative words	0.32 (0.023)*	[0.05; 0.55]	Valence	− 0.30 (0.031)*	[− 0.53;− .0.03]
% Past words	0.36 (0.009)**	[0.10; 0.58]	Past	0.36 (0.010)*	[0.09; 0.58]
% Future words	− .0.06 (0.66)	[− .0.33; 0.22]	Future	− .0.07 (0.62)	[− .0.34; 0.21]
% 1st person pronouns	0.31 (0.028)*	[0.04; 0.54]	Self-focus	0.19 (0.19)	[− 0.09; 0.44]
			% Internal	0.18 (0.20)	[− 0.10; 0.44]
			% External /Perceptually-coupled	− 0.16 (0.26)	[− 0.42; 0.12]
			% interoceptive	− .0.3 (0.37)	[− 0.39; 0.15]

Dynamics					
Duration	Partial r (p)	CI_95_	Affective transition probability	Partial r (p)	CI_95_
Total word count	− 0.29 (0.041)*	[− 0.52;− .0.01]	Positive to positive	− 0.31 (0.025)*	[− .0.54;− .0.04]
Total # of thoughts	− .0.12 (0.42)	[− .0.38; 0.17]	Positive to neutral	0.18 (0.21)	[− .0.09; 0.44]
Total # of strong transitions	− .0.13 (0.36)	[− .0.39; 0.15]	Positive to negative	− .0.01 (0.96)	[− .0.05; 0.27]
Total # of associative transitions	0.01 (0.93)	[− 0.26; 0.29]	Neutral to positive	− 0.04 (0.78)	[− .0.31; 0.24]
Duration _thought_	0.00 (0.98)	[− 0.28; 0.27]	Neutral to neutral	− .0.02 (0.92)	[− 0.29; 0.26]
Duration _positive thoughts_	− 0.27 (0.051)^**†**^	[− .0.51; 0.00]	Neutral to negative	0.05 (0.73)	[− 0.32; 0.23]
Duration _neutral thoughts_	− .0.08 (0.56)	[− 0.35; 0.20]	Negative to positive	− 0.32 (0.021)*	[− 0.55; − .0.05]
Duration _negative thoughts_	0.36 (0.010)*	[0.09; 0.58]	Negative to neutral	0.15 (0.30)	[− .0.13; 0.41]
			Negative to Negative	0.09 (0.53)	[− .0.19; 0.36]

Partial correlations between trait brooding scores and all content and dynamic variables of interest are shown. Partial correlations controlled for perceived censorship and daily thought similarity. For measures of duration, they also controlled for total word count. MNW = Mean Number of Words. Highlighted in bold are statistically significant partial correlations at p < .05. **P < .01, *P < .05, † < .06.

#### High brooding scores were associated with longer negative and shorter positive thoughts

Collapsing across affective content, dynamic indices did not predict individual differences in trait brooding. Trait brooding was unrelated to the total number of thoughts, the number of strong transitions, the number of associative transitions, or the mean number of words of thoughts (all *p*’s > 0.36). However, as trait brooding scores increased, participants produced fewer words overall across the 10-min TAP (*partial correlation* = − 0.29, *P* = 0.041).

Nevertheless, relationships with trait brooding became apparent when separating thoughts by their affective content. MNW_negative_ was significantly positively associated with brooding scores (*partial correlation* = 0.36, *P* = 0.010), and MNW_positive_ was numerically but non-significantly negatively associated with brooding (*partial correlation* = − 0.27, *P* = 0.051). MNW_neutral_ bore no relationship with brooding scores (*partial correlation* = − 0.08, *P* = 0.56).

Since the duration of positive and negative thoughts was not strongly correlated (*r* = 0.11, *P* = 0.44), we included both measures in the same model predicting trait brooding. The model explained 28.4% (*adjusted r*^2^) of the variance in brooding scores (*F*(5,45) = 4.96, *P* = 0.001), and both dynamic variables uniquely contributing (partial correlations similar to those reported Table 3). Overall, as brooding scores increased, positive thoughts became shorter and negative thoughts became longer.

#### Trait brooding tends to repel positive conceptual states: Affective transition probability analyses

**Figure 2:**
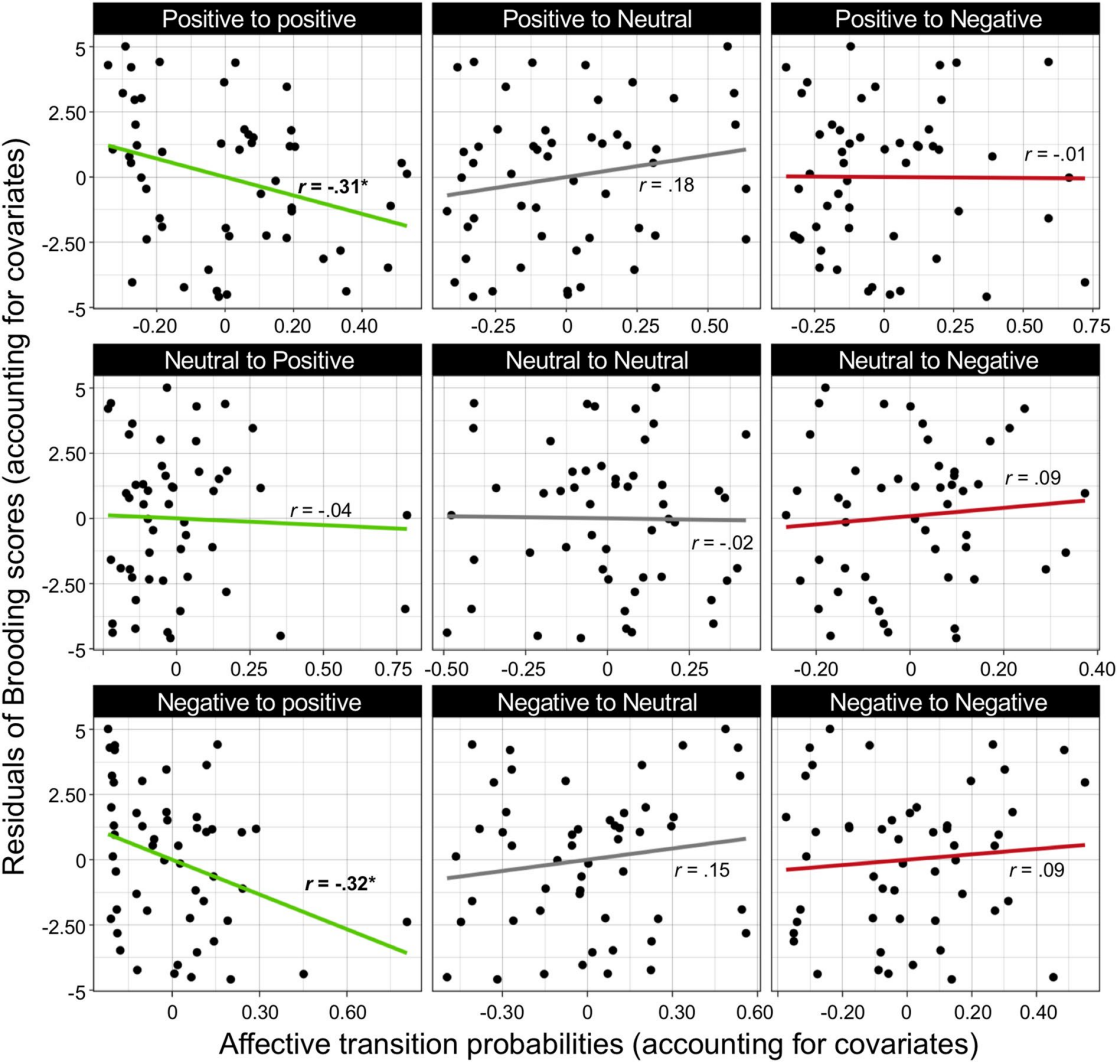
Linear relationships between affective transition probabilities and trait brooding. The probabilities of transitioning to a positive (green), neutral (grey), or negative (red) thought from current thoughts that are positive (top), neutral (middle), or negative (bottom) are examined in relationship to trait brooding. The y-axis reflects brooding once controlling for the covariates of censorship and daily thought similarity. *P < .05.

Higher brooding was associated with a lower likelihood of remaining in subsequent positive conceptual states (Pos → Pos: *partial r* − 0.31, *P* = 0.025) and a lower likelihood of transitioning from a negative thought to a positive thought (Neg → Pos: *partial r* = − 0.32, *P* = 0.021) (Fig. 2). Contrary to expectations, trait brooding was not associated with a stronger tendency to remain in a negative conceptual state following a negative thought (Neg → Neg: *partial r* = 0.09, *P* = 0.53). All other transition types were unrelated to brooding scores (all *P*’s > 0.21).

#### Brooding moderates the relationship between negative thought and average subsequent semantic similarity

In contrast to Study 1, the relationship between adjacent similarity and valence was not statistically significant, *b* = − 0.092, *χ*^*2*^(1) = 0.166, *P* = 0.68, *ß* = − 0.01 (*CI*_*95*_: [− 0.06; 0.04]). However, replicating the results from Study 1, average subsequent similarity was significantly negatively related to valence, *b* = − 0.766, *χ*^*2*^(1) = 5.87, *P* = 0.015, *ß* = − 0.07 (*CI*_*95*_: [− 0.13; − 0.01]).

We then constructed two regression models to assess if the relationship between thought similarity and valence was moderated by brooding scores. Similarity (adjacent or average subsequent, respectively), brooding score, and their interaction term were included as fixed effects, with a random effect (intercept) of participant. Both models controlled for individual differences in perceived thought censorship, similarity with daily life, and total word count.

**Figure 3:**
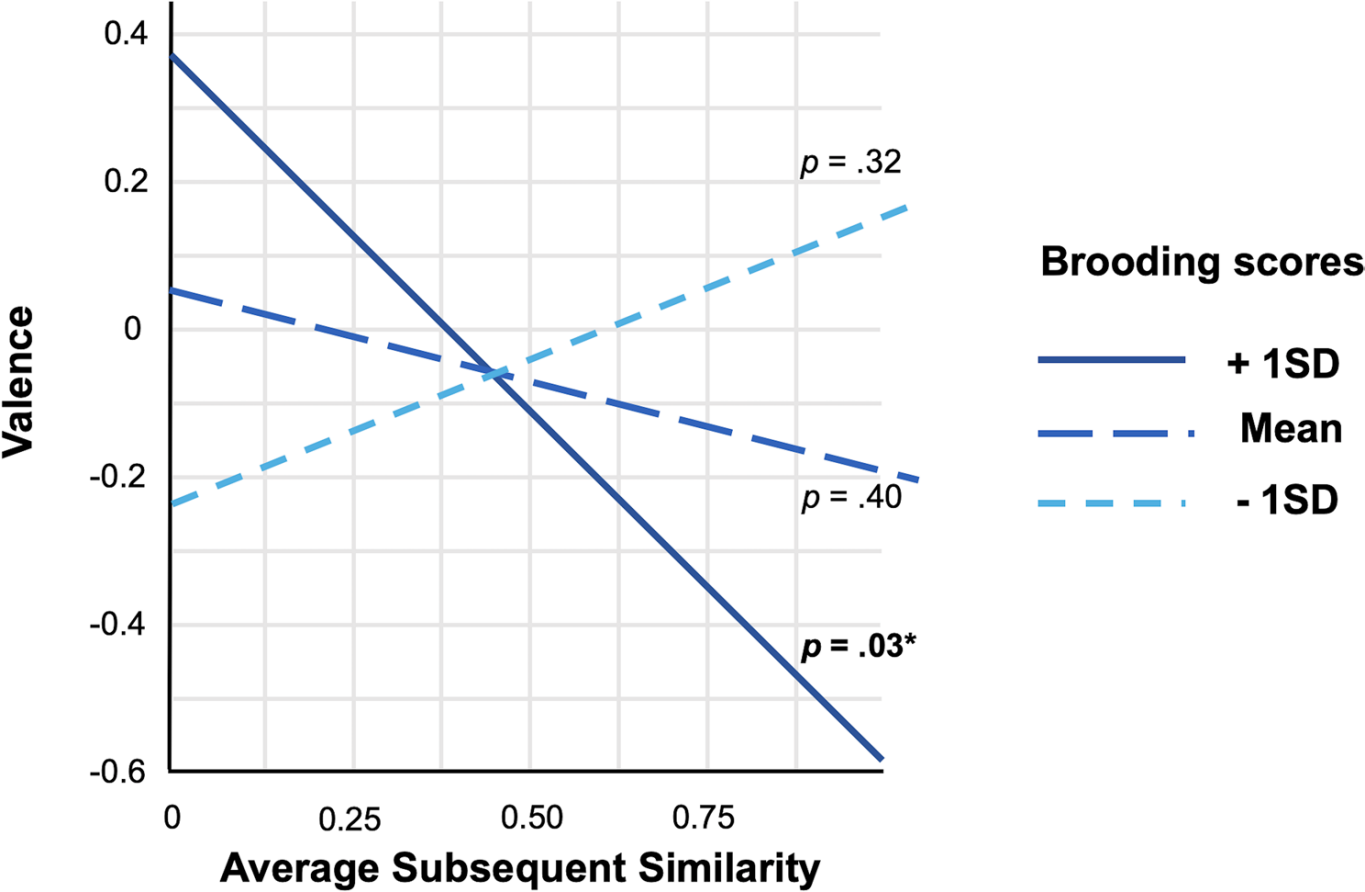
Brooding scores moderate the relationship between thought valence and average semantic similarity across subsequent thoughts. More negatively-valenced thoughts led to greater average subsequent semantic similarity (narrower conceptual scope) for individuals with higher brooding tendencies*.* The simple slopes relationships between valence and average subsequent semantic similarity for low and mean brooding groups was not significant. *P < .05.

We observed a significant interaction between brooding and subsequent similarity (Fig. 3), *b* = − 0.26, *χ*^*2*^(1) = 5.89, *P* = 0.015, suggesting that brooding scores moderated the relationship between valence and similarity over time. For those higher in brooding, greater negative valence was associated with more conceptual similarity over time, consistent with a progressive narrowing of conceptual scope (Fig. 3). Simple slopes analyses revealed that subsequent similarity was significantly related to valence when brooding scores were high (+ 1 SD above the mean), *b* = − 1.05, *P* = 0.030, whereas the same relationship was not significant for participants at the mean or − 1 SD below the mean (*P*’s = 0.32 and 0.40, respectively). The interaction was not significant for adjacent (*b* = − 0.137, *χ*^*2*^(1) = 3.05, *P* = 0.08), suggesting that this relationship is stronger when considering how thoughts unfold over time and not simply accounting for a single thought transition. Finally, we repeated both models without the covariates (similarity with daily life, censorship, and similarity total word count) and the pattern of significant results was consistent.

#### Thought content and dynamics account for unique variance in predicting trait brooding

In our nested model comparisons (Table 4), valence and % past-orientation were included as content predictors, and MNW_positive_, MNW_negative_, and affective transition probabilities Pos → Pos and Neg → Pos were included as dynamic indices. All models were significant (all *p*’s < 0.02). Content, censorship and similarity to daily life (Model 1) collectively explained 17.73% of the variance in trait brooding, and the addition of total word count (Model 2) explained significantly more variance than the initial model (*χ*^*2*^(1) = 7.21, *P* = 0.010, *Δadjusted r*^*2*^ = 8.35%, *ΔAIC* = 4.6). Adding dynamic predictors in Model 3 explained 10% more variance in brooding than Model 2 (*χ*^*2*^(4) = 2.83, *P* = 0.037, *Δadjusted r*^*2*^ = 10.36%, *ΔAIC* = 4.4), explaining a total of 36.44% of variance in brooding from a ten-minute TAP.

**Table 4:** Nested model comparisons of content and dynamic models predicting brooding scores.

Nested Model Comparisons (Manually-coded content)								
	AIC	r2	Adj. r2	*P*	SS	∆SS	*F*(df)	*P*
Model 1 (Content + Covariates)	251	24.31%	17.73%	0.011*	323.93			
Model 2 (add Total Word Count)	246.4	33.48%	26.08%	0.002**	284.72	39.21	7.21 (1)	0.010*
Model 3 (add Dynamics)	242	47.88%	36.44%	< 0.001***	223.08	61.64	2.83 (4)	0.037*

Nested Model Comparisons (LIWC-coded content)								
	AIC	r2	Adj. r2	P	SS	∆SS	*F*(df)	P
Model 1 (Content + Covariates)	247.8	31.65%	24.03%	0.003**	292.54			
Model 2 (add Total Word Count)	243.6	39.45%	31.20%	< 0.001***	259.14	33.39	6.29 (1)	0.016*
Model 3 (add Dynamics)	241.5	50.38%	37.98%	< 0.001***	212.36	46.78	2.2 (4)	0.086

Nested model comparisons assessed the explained variance and overall fit of three models predicting brooding scores. Model 1 for manually-coded content (top) included the significant predictors: valence, % past-orientation, % internal-orientation, censorship, and similarity to daily life. Model 1 for LIWC-coded content included % negative words, % past-oriented words, % first-person pronouns, censorship and similarity to daily life. Model 2 was identical to Model 1 but also included the third covariate of total word count. Model 3 included the same variables as Model 2 as well as mean number of words for positive thoughts, the mean number of words for negative thoughts, and affective transition probabilities from Pos → Pos and Neg → Pos.

*AIC* Akaike Information Criterion, *r*2: r squared, *adj.* r square: adjusted r square, *df* degree of freedom, *SS* sum of squares, ∆*SS* variation of sum of squares. Highlighted in bold are statistically significant models at p < .05. *P < .05, **P < .01, ***P < .001.

A similar analysis was performed using the LIWC-derived significant predictors (i.e., % of negative, past-oriented, and first-person pronoun words). Again, all models were significant (all *P*’s < .0.003). Though Model 2 explained significantly more variance than Model 1 (*χ*^*2*^(1) = 6.29, *P* = 0.016, *Δadjusted r*^*2*^ = 7.17%, *ΔAIC* = 4.2), the increase in variance explained by the Model 3 was not significantly greater than the intermediate model (*χ*^*2*^(4) = 2.20, *P* = 0.086, *Δadjusted r*^*2*^ = 6.78%, *ΔAIC* = 2.1).

### Study 2 discussion

Metrics derived from manual and LIWC coding of participants’ thoughts were remarkably similar across both studies. Thoughts sampled during an extended period of rest were rated as ecologically valid and showed similarity in content to studies reported in the literature using retrospective and experience sampling methods. On average, thoughts were neutral in valence^49,58^, and internal thoughts had a prospective bias^52^. Consistent with prior work suggesting that negative thinking is associated with more inflexibility^16,19,22–24,59^, negative thoughts further narrowed the conceptual scope of subsequent thoughts. This successful cross-validation provides a strong argument for the reliability of the task.

Applying the TAP to investigate brooding tendencies in young adults, higher brooding scores were associated with more negative, past oriented, and self-referential language. Those results mimic those found in clinically and subclinically depressed individuals^60,61^. As brooding increased, negative thoughts became longer and positive thoughts became shorter, and brooding tended to repel participants away from positive thoughts. Content and dynamic indices explained independent variance toward predicting brooding, and together accounted for more than a third of the adjusted *r*^2^ variability in trait brooding. These different results support the ecological, convergent, and clinical validity of the TAP.

## General discussion

Across two studies in independent samples of young adults, we audio recorded streams of consciousness during a 10-minute resting state paradigm and quantified metrics of content, dynamics, conceptual scope, and overall task experience. Study 1 provided quantitative benchmarks for these metrics and revealed the paradigm’s strong ecological validity. Study 2 replicated these metrics and related them to brooding scores, offering a cognitive signature of trait brooding during idle time. Collectively, these findings have important clinical and basic science implications and open up a range of exciting possibilities for future research.

### High brooding tendencies associate with altered thought content and dynamics at rest

Although dysfunctional content, dynamics, and conceptual scope have been recognized as central to many mental health disorders^4–6,10,24^, metrics beyond content have rarely been quantitatively explored, much less together in a single study. Similar to our study, Sripada and Taxali^41^ asked participants to “think aloud” for 30 min at rest and subsequently isolated individual thoughts. Although the authors did not extend their findings to individual differences in mental health, the stream of consciousness in healthy individuals tended to “clump” together by semantically similar content and then “jump” to different topics. Though our coding scheme differed, we similarly observed a clump-and-jump tendency, as suggested by the predominance of strong transitions (~ 73%). Additionally, Molina and colleagues^51^ used a TAP to examine changes in coarse linguistic content and topic shifting from a baseline period to a worry-prompted state across anxious, depressed, and healthy adults. Compared to the unprompted TAP, the worry TAP was characterized by more negative content and less shifts from topic to topic, although no group differences were observed. Relatedly, following a ruminative prompt, Lyubomirsky and colleagues^62^ found that dysphoric participants have a stronger tendency to reflect repeatedly on their problems. Here we extend beyond these prior studies by showing that in the absence of an overt mood induction or prompt, individuals with ruminative tendencies generate continuous thoughts that differ in both content and dynamics.

Our observation of restricted thought dynamics in individuals prone to brooding is consistent with a recent theoretical proposal that rumination is a form of thought constrained by a strong automatic affective pull^10,11^. Instead of freely moving from topic to topic, individuals prone to rumination exhibited longer negative thoughts, and negative content in ruminative individuals was linked to a stronger narrowing in conceptual scope over time, as suggested by higher levels of semantic similarity. Importantly, these dynamic indices explained variability in trait brooding beyond content and overall task experience. As such, they also converge with a recent study using a chained free association task showing that the conceptual associations of individuals with higher trait rumination were more strongly attracted to negative conceptual spaces and more likely to remain there longer^59^. Collectively, these studies provide behavioral support for the role of automatic constraints in restricting thought flexibility^10,14^, and are worthy of further empirical exploration given their predictive validity.

Although the overall number of thoughts and transitions did not differ across brooding scores, higher trait brooding was associated with restricted dynamics for negative compared to positive thoughts. These findings are broadly consistent with attentional scope models such as the Broaden-and-Build theory^22,23^ and aspects of the Attentional Scope Model of Rumination^24^ (see also^63^) and relate to prior findings showing that negative mood is associated with decreases in divergent thinking^64^. Negative thoughts were associated with a further narrowing of conceptual scope, and this relationship became stronger with increased trait brooding. Unlike extended predictions of the Attentional Scope Model of Rumination^24^, however, this narrowing of conceptual scope did not extend to positive thoughts in ruminative individuals. Additionally, the affective transition probability analysis did not suggest a propensity to stick to a given thought valence, with positive-to-positive transitions being significantly less likely as brooding scores increased. These findings contrast with the emotional inertia literature^20^, although emotional inertia pertains to the evolution of state affect^19^, while our results consider the evolution of affective content of thought and semantic similarity of content over time. Following from these findings, a speculative prediction would be that individuals high in trait brooding may perform particularly well on executive function tasks that require maintenance of attention towards negative stimuli, or switching from positively-valenced to negatively-valenced tasks. However, when such stimuli is no longer relevant for the task at hand, individuals high in brooding may exhibit difficulties disengaging from such stimuli or unrelated ruminative thought impairing task performance^65^.

We also observed that higher trait brooding associated with fewer words across the 10-min resting state paradigm. Although total word count was accounted for in our models of thought dynamics, the underlying cause of this lower word count in rumination remains unclear. We considered it may be related to higher censorship. Trait brooding was indeed marginally linked to higher thought censorship (*P* = 0.052), yet word count and censorship were not significantly correlated across participants (Study 1: *r* = − 0.20, *P* = 0.17; Study 2: *r* = − 0.13, *P* = 0.50), suggesting that higher brooding may be independently linked to both reduced overall thought fluency and somewhat greater censorship. Reduced word count in individuals with higher brooding could thus reflect difficulties in translating thoughts into spoken words (akin to verbal fluency), or an overall slowing of the rate of thought (i.e., slow ideation), both of which have been related to depression^66–68^.

### Thoughts emerging at rest are a clinically important yet empirically neglected aspect of mental life

Although the TAP can be used to capture thoughts during a task or following a prompt, here we capture thoughts emerging unprompted and at “rest,” when individuals are left alone with their thoughts with few opportunities for distraction. This aspect of our mental life requires more investigative effort for its potential relevance to mental health, as these moments may be most auspicious for the maladaptive aspects of thought^26–28^. Indeed, brooding being a state of high self-absorption associated with high level of decoupling from the external environment, it is conceivable that moments providing minimal opportunity for external engagement and distraction may act as contextual and mnemonic cues triggering habitual perceptually-decoupled brooding thought. It is also possible that negatively-valenced or self-related external stimuli can trigger the initiation of a chain of habitual ruminative thinking through associative processes^59,69^. How ruminative trains of thought begin is an important avenue for future investigation. Of relevance are mindfulness practices which train practitioners to face whatever thought content emerges with equanimity and to be comfortable being alone with their mind^70,71^. Additionally, the practice trains attentional focus^72^ by strengthening attentional control networks^73^, such that experienced meditators are more resilient to distraction and intrusive thoughts. Accordingly, meditation experience has been linked to greater deactivation of the default mode network during a variety of meditation practices^74^, and mindfulness-based therapies have been shown to reduce symptoms of depression and anxiety^75,76^.

These findings also provide insight into the cognitive mechanisms underpinning the clinical predictive validity of resting state functional MRI connectivity paradigms^77,78^. While the majority of resting state studies do not assess the content of thought emerging at rest, our findings suggest that the content and dynamics of thought explain a large amount of variability in ruminative traits between individuals. A prediction of our work is that this variability may also contribute to differences in resting state connectivity patterns between individuals, as well as variability within individuals, as suggested by recent studies^42,79–84^. For example, Turnbull and colleagues showed that as individuals perform an external task, task positive brain networks progressively decrease their activity with the passage of time, and individual differences in these neural dynamics track individual differences in self-reported task-unrelated thought^83^. Additionally, variation in neural dynamics across individuals have been linked to individual differences in fluid intelligence and divergent thinking, as well as one’s ability to regulate the emergence of task-unrelated thoughts based on task demands^84^. Overall, we suggest the think aloud paradigm may complement and extend this more recent research by affording a more precise mapping of thought content to the dynamics of hemodynamic activity.

### Advantages of the think aloud paradigm to study the stream of consciousness

As demonstrated here and by others^85,86^, the TAP is a short, convenient, and ecologically-valid paradigm with convergent and predictive validity that significantly adds to our understanding of thought content and dynamics. Additionally, it has the advantage of circumventing issues related to retrospective and subjective assessment of thought, which is common in resting state paradigms. Our findings using automated LIWC text analysis software also highlight the potential scalability of the TAP. Though manual ratings are considered more trustworthy, they are time intensive and require multiple raters. Automated and manual methods of assessment yielded similar content across Study 1 and 2 (*r* ≅ 0.50), and the Pearson and partial correlations with brooding scores were also quite similar, except for the manually coded level of self-focus of thought. The latter may be explained by the possibility that self-references and self-focus may tap into slightly different constructs. Although the time-consuming nature of our manual coding procedure limited our sample sizes in Study 1 and 2, future research would be benefitted by applying linguistic software such as LIWC to examine think aloud transcripts in much larger samples.

### Study limitations and future directions

This study has some limitations. Aside from being a predominantly young, educated, and relatively small sample, the laboratory environment in which the study took place may have felt unusual to the participants and prompted some levels of inhibition, affecting the quantity and content of thought produced. Additionally, we did not use a social desirability scale to determine whether participants’ answers to the ecological validity questions (i.e., censorship and similarity to daily life) were sincere. However, multiple findings including thought fluency analyses, as well as the presence of content including self-disclosure, self- or other-focused criticism, cursing and so on converge to suggest that participants’ transcripts were largely sincere.

Another limitation is that we did not assess state affect during the experiment and thus could not examine the effect of mood on our findings. Additionally, our participants were skewed towards exhibiting lower levels of brooding, although the range in scores spanned nearly the full scale. A final limitation is that many of our statistical effects are relatively modest and would likely not survive corrections for multiple comparisons, especially considering our relatively small sample sizes. However, findings pertaining to variables other than rumination replicated across two studies, were consistent across measurement techniques (manual and LIWC), and were predicted a priori (with the exception of the duration analyses for which we also acknowledged an alternative hypothesis). Nonetheless, we call for future research to replicate our findings in larger sample sizes, including in clinically-diagnosed samples.

An important avenue for future work will therefore be to extend the TAP to clinical samples with higher levels of rumination. From the intervention side, comparing the content of thoughts prior, during, and after clinical interventions may also be an avenue of interest^87^, with potential to illuminate the evolution of the stream of consciousness as symptoms change over time. Despite these limitations, the TAP is a promising paradigm with basic science, clinical and therapeutic relevance.

## Supplementary Information

Supplementary Information.

## Data Availability

The data that support the findings of this study are available from the corresponding author upon reasonable request.
