# Supplementary material for: The think aloud paradigm reveals differences in the content, dynamics and conceptual scope of resting state thought in trait brooding
Source: Sci Rep. 2021 Sep 30;11:19362. doi: 10.1038/s41598-021-98138-x (PMC8484343; doi:10.1038/s41598-021-98138-x)
Supplement: Supplementary file 1 — Supplementary Information. [file 41598_2021_98138_MOESM1_ESM.pdf]

SUPPLEMENTAL MATERIALS:

The think aloud paradigm reveals differences in the content, dynamics and conceptual scope of resting state thought in trait brooding

Quentin Raffaelli [1], Caitlin Mills [2], Matthias R. Mehl [1], Nadia-Anais de Stefano [1], Kate Chambers [1], Surya A. Fitzgerald [1], Ramsey Wilcox [1], Kalina Christoff [3,4,5], Eric S. Andrews [1], Matthew D. Grilli [1,6], Mary Frances O'Connor [1], & Jessica R. Andrews-Hanna [1,7,8]

[1] Department of Psychology, University of Arizona, Tucson, AZ, USA
[2] Department of Psychology, University of New Hampshire, Durham, NH, USA
[3] Department of Psychology, University of British Columbia, Vancouver, British Columbia, Canada
[4] Centre for Brain Health, University of British Columbia, Vancouver, British Columbia, Canada
[5] Peter Wall Institute for Advanced Studies, University of British Columbia, Vancouver, British Columbia, Canada
[6] Department of Neurology, University of Arizona, Tucson, AZ, USA
[7] Cognitive Science, University of Arizona, Tucson, AZ, USA
[8] Evelyn F. McKnight Brain Institute, University of Arizona, Tucson, AZ, USA

Contact:

Quentin Raffaelli: quentinraffaelli@email.arizona.edu

Department of Psychology, 1503 E University Blvd., Tucson, AZ 85721

Jessica R. Andrews-Hanna: jandrewshanna@email.arizona.edu

Department of Psychology, 1503 E University Blvd., Tucson, AZ 85721

Think aloud instructions

For this task, we want you to describe your stream of consciousness. It consists of you simply voicing out loud whatever comes to your mind. At times, your attention might be oriented outwards, towards the sights and sound of your external environment. At other times, you may be thinking about a particular topic, or your attention may be oriented towards your feelings, bodily sensations, or emotions. Regardless of what comes to mind at different moments in time, we would like you to continuously relay that information out loud.

Please know that no one outside the room will be able to hear what you say. The person that will transcribe the audio will not know your name and will not be the experimenter (i.e. Me) that was present during the experimental session.

In summary, we ask that you remain awake and simply voice out loud whatever comes to your mind.

Any questions?

	Study 1	Study 2
Gender		
Female	13	32
Male	12	18
Trans	0	0
Other	0	1
NA	2	0
Race		
White	15	32
Black/African American	1	1
Asian/Asian American	5	2
Native/Pacific islander	0	1
American Indian/Alaska	0	2
Prefer not to respond	2	9
Multiracial	2	4
NA	2	0
Ethnicity		
Latino/hispanic	5	20
Not latino /hispanic	20	29
Prefer not to respond	0	2
NA	2	0
Education		
Less than high school	0	0
High school/GED	7	16
Some college	16	32
2 year college degree	1	1
4 year college degree	1	1
Master	0	1
PhD	0	0
Professional degree	0	0
NA	2	0

Supplementary Table S1. Demographics of participants for Study 1 and 2. Gender, race, ethnicity, and educational information about the sample in Study 1 and 2. The NA in Study 1 are a results of failure of collection of demographics information for 2 participants.

	Study 1	Study 2
Total # of thought	.89, CI_{95} = [.72;.95]	.79, CI_{95} = [.54;.89]
Duration $_{thought}$	.60, CI_{95} = [.42;.75]	.74, CI_{95} = [.63;.83]
Total # of strong transitions	.96, CI_{95} = [.94; 98]	.93, CI_{95} = [.88; 95]
Total # of associative transitions	.28, CI_{95} = [.07;.50]	.35, CI_{95} = [.11;.55]
Valence	.66, CI_{95} = [.63;.69]	.67, CI_{95} = [.64;.69]
Self-focus	.77, CI_{95} = [.73;.80]	.76, CI_{95} = [.69;.80]
Temporal orientation	0.63	0.68
Perceptual orientation	0.79	0.81

Supplementary Table S2. Inter-rater reliability between Study 1 and 2. For all variables, we calculated the Inter-class correlation (ICC) with 95% confidence interval, except for perceptual orientation and temporal orientation for which Cohen's Fleiss and Kappa, respectively, were calculated instead.

Content			
LIWC	***partial r (p)***	***Manual coding***	***partial r (p)***
% Positive words	-.14 (.33)		
% Negative words	**.33 (.02)***	Valence	-.26 (.07)
% Past words	**.38 (.006)****	Past	**.36 (.01)***
% Future words	-.06 (.68)	Future	-.10 (.48)
% 1st person pronouns	**.39 (.005)****	Self-focus	.21 (.14)
		% Internal	.20 (.16)
		% External / Perceptually-coupled	-.18 (.20)
		% interoceptive	-.12 (.41)
**Dynamics**			
Duration	***partial r (p)***	***Markov chain***	***partial r (p)***
Total word count	**-.30 (.03)***	Positive to positive	-.25 (.08)
Total # of thoughts	-.08 (.57)	Positive to neutral	.21 (.15)
Total # of strong transitions	-.03 (.82)	Positive to negative	.01 (.97)
Total # of associative transitions	-.13 (.35)	Neutral to positive	.13 (.39)
Duration $_{thought}$	**-.30 (.035)***	Neutral to neutral	-.03 (.83)
Duration $_{positive\ thought}$	**-.31 (.027)***	Neutral to negative	.19 (.21)
Duration $_{neutral\ thought}$	-.16 (.29)	Negative to positive	**-.29 (.04)***
Duration $_{negative\ thought}$	.16 (.27)	Negative to neutral	.15 (.31)
		Negative to Negative	.07 (.62)

Supplementary Table S3. Thought content and dynamic correlates of trait brooding. Pearson Correlation between brooding scores and its thought content and dynamic predictors. For measures of duration, they also controlled for total word count. MNW = Mean Number of Words. **<.01, *p < .05.

Markov chain transition	Mean	SD	Median
Positive to positive	29%	25%	29%
Positive to neutral	41%	30%	33%
Positive to negative	30%	28%	24%
Neutral to positive	23%	21%	17%
Neutral to neutral	48%	24%	48%
Neutral to negative	29%	19%	28%
Negative to positive	21%	20%	19%
Negative to neutral	47%	28%	45%
Negative to negative	32%	24%	31%

Supplementary Table S4. Descriptive statistics for the affective transition probabilities between the different valence states. Mean, SD, and median of transition probabilities for all possible transitions.
